# Serum supplemented culture medium masks hypertrophic phenotypes in human pluripotent stem cell derived cardiomyocytes

**DOI:** 10.1111/jcmm.12356

**Published:** 2014-07-01

**Authors:** Cheryl Dambrot, Stefan R Braam, Leon G J Tertoolen, Matthew Birket, Douwe E Atsma, Christine L Mummery

**Affiliations:** a Department of Anatomy and Embryology, Leiden University Medical CenterLeiden, The Netherlands; b Department of Cardiology, Leiden University Medical CenterLeiden, The Netherlands; c Pluriomics BVLeiden, The Netherlands

**Keywords:** human pluripotent stem cells, hypertrophy, serum, cardiomyocytes

## Abstract

It has been known for over 20 years that foetal calf serum can induce hypertrophy in cultured cardiomyocytes but this is rarely considered when examining cardiomyocytes derived from pluripotent stem cells (PSC). Here, we determined how serum affected cardiomyocytes from human embryonic- (hESC) and induced pluripotent stem cells (hiPSC) and hiPSC from patients with hypertrophic cardiomyopathy linked to a mutation in the MYBPC3 gene. We first confirmed previously published hypertrophic effects of serum on cultured neonatal rat cardiomyocytes demonstrated as increased cell surface area and beating frequency. We then found that serum increased the cell surface area of hESC- and hiPSC-derived cardiomyocytes and their spontaneous contraction rate. Phenylephrine, which normally induces cardiac hypertrophy, had no additional effects under serum conditions. Likewise, hiPSC-derived cardiomyocytes from three MYBPC3 patients which had a greater surface area than controls in the absence of serum as predicted by their genotype, did not show this difference in the presence of serum. Serum can thus alter the phenotype of human PSC derived cardiomyocytes under otherwise defined conditions such that the effects of hypertrophic drugs and gene mutations are underestimated. It is therefore pertinent to examine cardiac phenotypes in culture media without or in low concentrations of serum.

## Introduction

Human pluripotent stem cells (hPSC), particularly those derived as induced pluripotent stem cells (hiPSC) from patients with genetic diseases, are increasingly regarded as useful for disease modelling and drug target discovery. hiPSC are considered particularly valuable because they capture the genome of the individual from whom they are derived. They have the ability to self-renew over long periods and differentiate into all cell types of the body and thus represent a permanent resource for modelling human disease. Although directed differentiation is still challenging for many lineages, cardiomyocytes were among the first functional cells to be derived and characterized from human embryonic stem cells (hESC) [1–3] and they can now be generated efficiently from hiPSC using similar protocols. Cardiomyocytes derived from human (h)PSC have been successful in mimicking reported drug responses in patients [4–7] including the positive chronotropic response to phenylephrine (PE), an α-adrenergic agonist, and isoproterenol (ISO), a β-adrenergic agonist [3]. These adrenergic stimuli have also been shown to induce pathological hypertrophy in cultured cardiomyocytes [8–10]. Pathological hypertrophy is an abnormal increase in cell size accompanied by the re-expression of foetal cardiac genes [11]. Apart from drug-induced cardiac hypertrophy, cardiomyocytes from hiPSC of patients with mutations associated with hypertrophic cardiomyopathy (HCM) also show features of hypertrophy and are thus potentially useful disease models [12]. Cardiomyocytes from patient hiPSC have already increased our knowledge of cardiac diseases [12–14], complementing and extending previous work based on clinical data and primary human cardiomyocyte cultures.

While the uses of hPSC-derived cardiomyocytes are becoming well-established, there is still no single method to derive these cells in culture. Although differentiation protocols are increasingly based on defined media and timed growth factor addition [15–18], foetal calf serum (FCS) is still often present, either during differentiation or during long-term maintenance to support survival as the cardiomyocytes attain some degree of maturity. Of 119 articles published using hPSC-CM in 2012 and 2013, 54 used 5% or higher concentrations of serum in the differentiation and/or maintenance medium. In others serum was present but at concentrations ≤2%. Since the exact composition of serum is unknown and varies considerably from batch to batch [19], its presence can confound interpretation of experiments investigating disease phenotype or drug responses. For example, it has long been known that serum or serum deprivation can profoundly affect primary neonatal rat cardiomyocytes (Rat-CM) in culture [20–24]. Cell size and protein-to-cell ratio can increase and additional stress-fibre-like structures may be induced in response to serum, which is indicative of cardiac hypertrophy [21,24]. Other studies have used serum as a hypertrophic stimulus for cardiomyocytes [20,25,26], in place of the more commonly used hypertrophic stimuli PE or ISO [10]. The impact of serum on the cardiomyocyte phenotype and, more specifically, on the manifestation of the disease in the case of cardiomyocytes derived from patient hiPSC, remains unreported.

Here, we examined the effects of serum and PE in the widely used Rat-CM model and confirmed findings reported previously. We then investigated the effects of serum on healthy- and HCM patient-derived hPSC-CM obtained under serum-free, defined conditions. Parameters examined included cell surface area, sarcomeric structure and beating frequency. These are common descriptors of phenotype in the literature. The effects of serum were compared with those induced by the hypertrophic drugs PE and ISO. We found that serum-induced increased cell surface area and beating frequency and disrupted sarcomeric structure, indicating a hypertrophic response in both hESC-CM and hiPSC-CM. This masked the disease-associated hypertrophy of HCM-hiPSC-CM that was evident in the absence of serum. These results have important implications for conditions under which disease phenotypes are examined.

## Material and methods

### Ethics statement

The isolation of neonatal rat cardiomyocytes was approved by the Animal Experiment Committee of Leiden University Medical Center and complies with the Guide for the Care and Use of Laboratory Animals as stated by the U.S. National Institutes of Health. Human skin biopsies were obtained from patients after individual written permission using standard informed consent procedures following approval for use in this study by Leiden University Medical Center's medical ethics committee; this conforms to the Declaration of Helsinki. Control skin samples were obtained as waste tissue from donors in accordance with the Dutch federation of Biomedical Scientific Societies ‘Use of human tissue for scientific research’ and ‘Code of good use’ directives. All samples were collected by the treating physician and then anonymized.

### Neonatal rat cardiomyocytes

Neonatal rat cardiomyocytes were isolated as previously described [27]. Briefly, rats were anaesthetized with inhaled isoflurane (4–5%) and adequate anaesthesia was confirmed by the absence of pain reflexes before excising the hearts. Ventricles from the dissected hearts were minced and dissociated using collagenase and DNase and then suspended in Ham's F10 medium (ICN Biomedicals, Eschwege, Germany) with 10% horse serum (Invitrogen, Leek, The Netherlands) and 10% FCS. To allow for preferential attachment and negative selection of the non-cardiomyocytes, the cell suspension was pre-plated for 1 hr in a tissue culture dish. The non-adherent cardiomyocytes were then collected and plated on Matrigel-coated coverslips (plating density of 20,000 cells/cm^2^) in isolation medium or serum-free BEL medium [Iscove's Modified Dulbecco's Medium (IMDM) supplemented with L-glutamine and 25 mM HEPES (Invitrogen), F12 Nutrient Mixture (HAM) supplemented with Glutamax (Invitrogen), 5% protein free hybridoma medium (Invitrogen), 0.25% deionized albumin from BSA (Sigma-Aldrich) in IMDM, 1% Chemically Defined Lipid Concentrate (Invitrogen), 0.1% Insulin-Transferrin-Selenium-X supplement (Invitrogen), 450 μM 1-Thioglycerol (Sigma-Aldrich), 5 mg/ml L-ascorbic acid 2-phosphate (Sigma-Aldrich), 1% Glutamax (Invitrogen), 25 U/ml penicillin, 25 μg/ml streptomycin (both Invitrogen)]. 12 hrs later the medium was replaced and the cells were maintained in the original isolation medium (20% serum), 5% FCS in BEL or serum-free medium BEL for 24 hrs before treatment with 100 μM PE for an additional 24 hrs.

### hESC culture

The Nkx 2-5^eGFP/w^ hESC line [28] was maintained as a single cell culture in DMEM/F12 (Invitrogen) containing 20% Knockout Serum replacement (Invitrogen), 1× MEM-Non-Essential amino acids (Invitrgoen) 0.1 mM 2-Mercaptoethanol (Invitrogen), 10 ng/ml Human basic fibroblast growth factor (Peprotech, Hamburg, Germany), 25 U/ml penicillin and 25 μg/ml streptomycin (both Invitrogen) and passaged every 3–4 days, as previously described [29].

### Human iPSC derivation

Human dermal fibroblasts were isolated from biopsies from a control subject and patients carrying a MYBPC3 gene mutation and displaying an HCM phenotype. Fibroblasts were reprogrammed to hiPSC as previously described [30]. hiPSC were routinely cultured on Matrigel (BD Biosciences, Leiden, The Netherlands)-coated tissue culture dishes in mTESR according to the manufacturer's protocol (Stem Cell Technologies, Vancouver, Canada). The cells were mechanically passaged weekly using 1 mg/ml Dispase (Gibco, Merelbeke, The Netherlands). Additional methods are given in supporting information.

LUMC0004iCtrl (Healthy), LUMC0033iMyBPC (HCM1), LUMC0034iMyBPC (HCM2), LUMC0035iMyBPC (HCM3) hiPSC were used in this study.

### Generation of cardiomyocytes

Cardiomyocytes were generated from the Nkx 2-5^eGFP/w^ hESC line [28] and hiPSC using a monolayer method in defined medium with timed addition of growth factors and small molecules. hESC were plated at a density of 10,000 cells/cm^2^ and hiPSC were dissociated into small clumps 3 days prior to differentiation and allowed to attach on Matrigel-coated dishes in mTESR (∼1.5 clumps/cm^2^) before starting the differentiation procedure. Differentiation was induced in low-insulin (1 mg/l), ‘BEL medium’ [16] plus 20 ng/ml Activin A (R&D Systems, Oxford, UK), 20 ng/ml BMP4 (R&D Systems) and 1.5 μM CHIR99021 (Axon, Gilze, The Netherlands). These factors were removed after 3 days and replaced with BEL plus 5 μM XAV939 (R&D systems) and Matrigel (1:100; BD Biosciences) to prevent cell detachment. The cells were refreshed with BEL 3 days later and subsequently maintained in BEL (changing medium twice a week). On average, the differentiation efficiency of this method is 28–44% over several hPSC lines as determined by flow cytometry for cardiac troponin T expression (cTnT) (Fig. S1).

### Cell stimulation and cell surface area

Cardiomyocytes were dissociated into single cells 20 days after the start of differentiation using TrypLE Select (Invitrogen) for 30 min. at 37°C and plated onto Matrigel-coated coverslips (25,000 cell/cm^2^) in serum-free BEL medium. After 10 days, the culture medium was placed with BEL containing 100 μM PE (Sigma-Aldrich), 100 nM ISO (Sigma-Aldrich), 5% serum, 20% serum [Greiner (Serum A) or Gibco (Serum B)], or left untreated for 72 hrs. After 72 hrs, all coverslips were fixed in 2% paraformaldehyde for 30 min. For long-term experiments, cells on coverslips were exposed to serum for 7 days followed by 72 hrs treatment ±PE prior to fixation. For reversibility experiments, cells were treated as above and subsequently refreshed with serum-free BEL medium for 3, 5 or 10 days.

For determination of cardiomyocyte cell surface area, fixed cells were labelled with anti-α actinin (1:800; Sigma-Aldrich) followed by Cy3 (ImmunoResearch, Sigma-Aldrich, Zwijndrecht, The Netherlands) secondary as previously described [31]. Nuclei were labelled with DAPI. For cell surface area measurements, 9–15 areas were randomly selected from each 10 mm coverslip and visualized using a Nikon Eclipse T*i*-S microscope (Amsterdam, The Netherlands). Single plane of 1200 × 1600 pixels images at 313 nm/pixel resolution were recorded using a Plan fluor 20×/0.50 lens and analysed using ImageJ software. Representative sarcomere images were visualized using a Leica TCS SP5 confocal microscope (Rijswijk, The Netherlands); single planes of 1024 × 1024 pixels images at 44 nm/pixel resolution were recorded using a Plan-Apochromat 40×/1.25 oil and 100×/1.4 oil lens. Sarcomeric structures of cardiomyocytes were divided into three categories, visible well-organized striations (+: category 1), some striations mostly disorganized (+/−: category 2) and poor or non-existent striations (−: category 3).

### Calcium imaging

Cell cultures undergoing cardiac differentiation were dissociated after 20 days using TrypLE Select and replated on 96-well Matrigel-coated imaging plates (BD Biosciences, 40,000 cells/cm^2^). To determine calcium transient/contraction frequency 13 days after plating, cells were loaded with 5 μM fluo-4am (Invitrogen) for 15 min. in the presence of 0.2% Pluronic in Tyrode's solution (140 mM NaCl, 10 mM glucose, 5 mM HEPES, 5.4 mM KCl, 1.2 mM MgCl_2_, 1.8 mM CaCl_2_, pH 7.4). The cells were then washed three times with Tyrode's solution and maintained in 100 μl of Tyrode's solution. With the plate maintained at 37°C, time-lapse of Fluo-4am fluorescence was recorded using a Leica AF6000 microscope equipped with a Hamamatsu EM-CCD camera. Contracting areas were identified and imaged consecutively for 1.4 min. (1500 images at 51 ms/cycle). After recording a baseline for 20 sec. drug or serum additions were made to achieve final concentrations of 100 nM ISO, 100 μM PE, 5% serum, 20% serum, or vehicle only. Calcium transient frequency before and after treatment was determined using ImageJ and Z-profile_ImJ software, tailored for use here to convert raw data to dF/F results [32].

### Statistical analysis

Results are expressed as mean ± SEM. Comparisons were made using one-way anova with Bonferroni's multiple comparison post-test. Values of *P* ≤ 0.05 were considered significant. Statistical analyses were performed with GraphPad Prism.

## Results

### A hypertrophic response to serum in neonatal rat cardiomyocytes

**Fig. 1:**
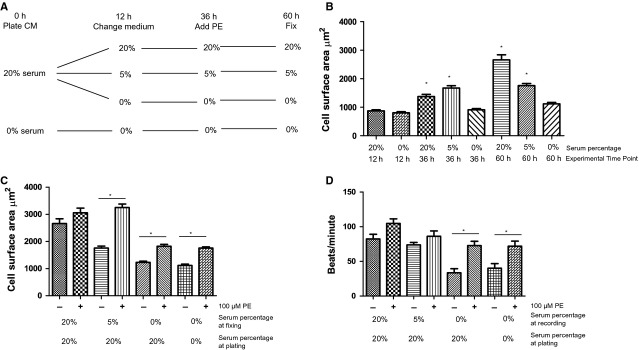
Responses of neonatal Rat-CM to serum. (**A**) Schematic timeline of treatment procedure. Percentages indicate the serum concentrations. In parallel controls, PE was omitted. (**B**) Cell surface area of Rat-CM in 20%, or 0% serum at 12, 36 and 60 hrs after plating and 5% serum 36 and 60 hrs after plating. *P < 0.05 20% serum (12 hrs) *versus* other conditions (*n* ≥ 67). (**C**) Cell surface area of neonatal Rat-CM (60 hrs) maintained in isolation medium (20% serum) throughout or subsequently reduced to 5% or 0% serum, or initially plated in 0% serum 100 μM PE for 24 hrs. *P < 0.05 untreated *versus* PE-treated (*n* ≥ 59 cells). (**D**) Beating frequency of Rat-CM (60 hrs) 100 μM PE. *P < 0.05 untreated *versus* PE-treated (*n* ≥ 7 measurements).

We first confirmed well-established hypertrophic effects of serum and PE on Rat-CM using cell surface area and beating frequency as standard surrogate measures. Rat-CM was plated in the original isolation medium containing 20% serum or in the absence of serum (Fig. 1A, 0 hr). After 12 hrs, the cells were transferred to 20%, 5% or 0% serum and maintained at this serum concentration until the end of the experiment (60 hrs). Although the initial plating of these cells with or without serum (12 hrs exposure) did not affect cell surface area (Fig. 1B), by 36 hrs, cells in serum (5% or 20%) were significantly larger than those in absence of serum (Fig. 1B). By 60 hrs, a concentration-dependent effect was evident with cells maintained in 20% serum now significantly the largest (Fig. 1C). No significant difference was observed in the sarcomeric structure or organization between the groups (Fig. S2).

Exposure of cardiomyocytes to PE has been previously shown to increase their cell surface area [33]. We observed a similar hypertrophic effect with exposure to PE for cells maintained in 0% or 5% but not 20% serum (Fig. 1C). It was further noted that Rat-CM beat significantly more slowly in the absence than in the presence of serum (Fig. 1D, *n* ≥ 7, *P* < 0.05). PE exerted an expected positive chronotropic action under serum-free conditions (Fig. 1D, *n* ≥ 7, *P* < 0.05), effects which were similar to serum. Cells maintained in serum were refractory to the positive chronotropic effect of PE.

### A hypertrophic and positive chronotropic response of hESC-CM to serum containing medium

**Fig. 2:**
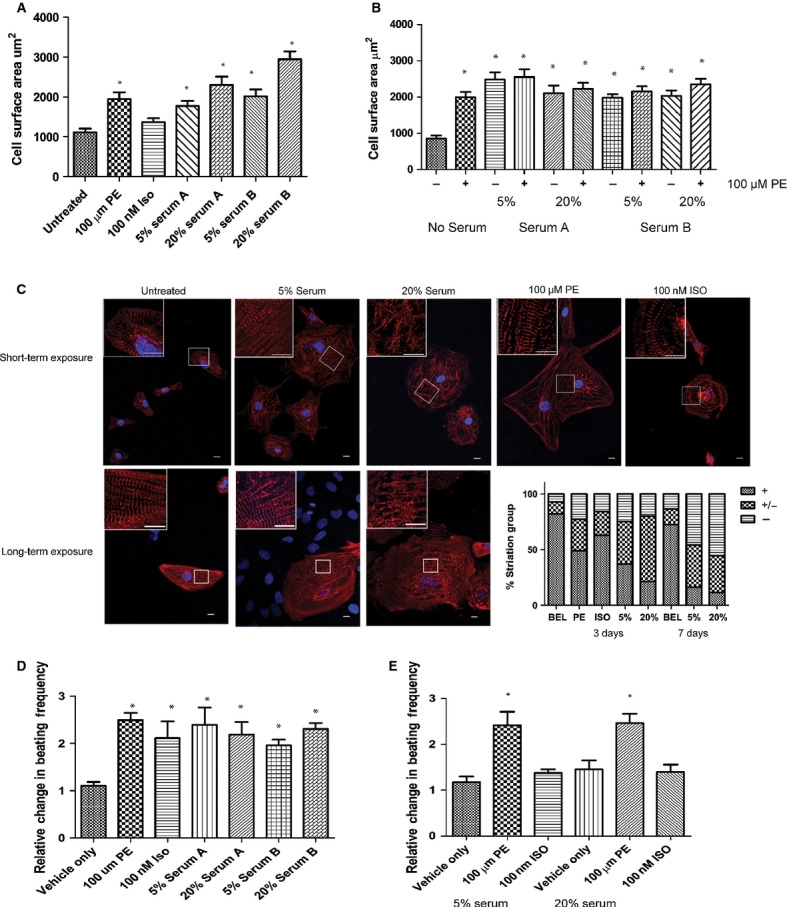
Response of hESC-CM to serum. (**A**) Cell surface area of hESC-CM treated with 100 μM PE, 100 nM ISO, 5% serum or 20% serum for 72 hrs compared to untreated *n* ≥ 72 cells. *P < 0.05 untreated *versus* treated. (**B**) Cell surface area of hESC-CM treated with 5% or 20% serum for 7 days 100 μM PE for 72 hrs. *P < 0.05 no PE added *versus* PE-treated (*n* ≥40). (**C**) Representative immunofluorescent images of hESC-CM treated with 100 μM PE, 100 nM ISO, 5% serum or 20% serum for 72 hrs (short-term exposure) or 5% serum and 20% serum for 7 days (long-term exposure). Stacked bar graph of the percentage of cells with sarcomeric striation (visible well-organized striations (+), some (disorganized) striations (+/−), and poor/non-existent striations (−)); *n* ≥ 72 cells. Red: α-actinin, Blue: DAPI, scale bar: 10 μm. (**D**) Relative change in beating frequency of hESC-CM after immediate addition of 100 μM PE, 100 nM ISO, 5% serum, 20% serum or vehicle only control. Results normalized to initial beating frequency. *P < 0.05 vehicle only *versus* treated (*n* ≥ 7) measurements. (**E**) Relative change in beating frequency of long-term serum treated hESCCM after immediate addition of 100 μM PE, 100 nM ISO or vehicle only control. Results normalized to initial beating frequency. *P < 0.05 vehicle only *versus* treated (*n* ≥5) measurements.

We assessed whether hESC-CM responded similarly to Rat-CM on exposure to serum or known adrenergic stimuli. When measured after 72 hrs, cells maintained in 5% or 20% serum [two batches, Sigma-Aldrich (Serum A) and Gibco (Serum B)] or PE all showed increased cell surface area (Fig. 2A). The β-adrenergic agonist ISO failed to induce a significant increase (Fig. 2A). Combined exposure of cells to both serum (>7 days) and PE (72 hrs) did not induce any additional increase in cell surface area (Fig. 2B).

As results for serum brands A and B were similar, only serum B was used in subsequent experiments. Interestingly, besides the increased cell surface area, hESC-CM exposed to 5% or 20% serum developed abnormal sarcomeric structures. These effects were quantified and cells were divided into three categories: (1) visible well-organized striations (+), (2) some (disorganized) striations (+/−) and (3) poor/non-existent striations (−). The percentage of cells in category 1 was reduced and in category 3 was increased (Fig. 2C) as early as 72 hrs after serum addition and was further exacerbated by long-term serum exposure (Fig. 2C).

Stimulation of the α-adrenergic system with PE or the β-adrenergic system with ISO has been shown previously to induce a positive chronotropic response in hESC-derived cardiomyocytes [3]. We confirmed these responses in this model. 100 μM PE induced a 2.25-fold relative increase in beating frequency (*n* = 11 beating clusters) and 100 nM ISO induced a 1.9-fold relative increase (*n* = 11 beating clusters; Fig. 2D). Furthermore, we found that serum addition to a serum-free culture caused a similar increase in beating frequency. Following chronic exposure to serum (>7 days) the positive chronotropic effect of PE was still evident but the effect of ISO was completely lost (Fig. 2E). Apart from altered beating frequency however, all other parameters of the calcium transient, such as upstroke velocity, slope decay and amplitude, showed no significant difference between treated and untreated groups (data not shown).

### The hypertrophic effect of serum on hESC-CM may be irreversible

**Fig. 3:**
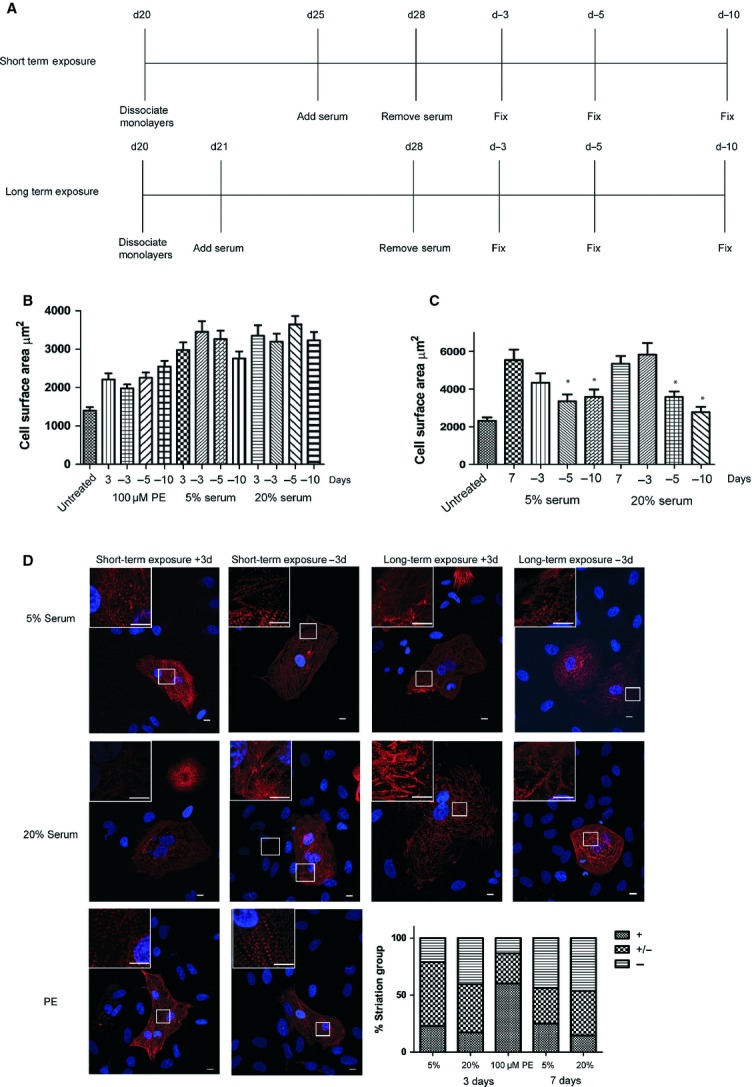
The stability of serum-induced alterations in hESC-CM. (**A**) Schematic of treatment protocol. Cell surface area of hESC-CM treated with 5% or 20% serum for 3 days (*n* ≥ 81) (**B**) or 7 days (*n* ≥ 42) (**C**) and then removed for 3, 5 or 10 days. *P < 0.05 serum *versus* -removal. (**D**) Representative immunofluorescent images of hESC-CM treated with aaa100 0M PE, 5%, and 20% serum for 72 hrs +3 days (short-term exposure +3 days) or 5% or 20% serum for 7 days +3 days (long-term exposure +3 days) and then removed for 3 days (short-term exposure-3d and long-term exposure-3d, respectively). Stacked bar graph of the percentage of cells of different sarcomeric structural class (visible well-organized striations (+; category 1), some (disorganized) striations (+/−; category 2), and poor/non-existent striations (−; category 3); *n* ≥ 72). Red: α-actinin, Blue: DAPI, scale bar: 10 μm.

To assess the stability of the serum- or PE-induced hypertrophy, we measured cardiomyocyte surface area upon withdrawal of these factors (Fig. 3A). For cells exposed to serum or PE for 72 hrs, there was no significant decrease in cell surface area at any time-point and the cells remained significantly larger than the untreated cells (Fig. 3B). However, cells treated with serum for 7 days and then deprived of serum showed a significant decrease in cell surface after 5 days in serum-free medium (Fig. 3C). By this time-point, these cells were no longer significantly larger than the untreated cells.

The sarcomeric structures that had already remodelled in the presence of serum appeared even more disrupted, with many more cells showing disorganized or missing striations (category 3) (Fig. 3D). This loss of sarcomeric structure was increased the longer the cells were deprived of serum (data not shown). The removal of PE from the cells had little or no effect on the sarcomeric structure (Fig. 3D).

### Serum effects on cardiac hypertrophic disease modelling

**Fig. 4:**
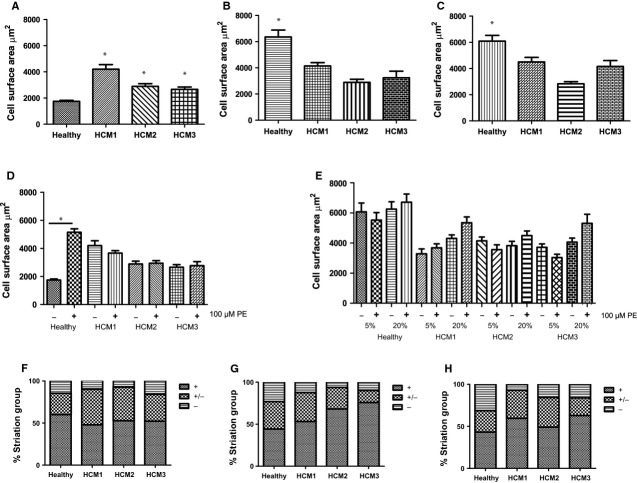
Response of hiPSC-CM to serum. Cell surface area of hiPSC-CM derived from a healthy individual (healthy) and three patients with hypertrophic cardiomyopathy (HCM1, HCM2, HCM3) in (**A**) serum-free medium *P = 0.05 healthy *versus* HCM*n* ≥ 60 cells. (**B**) Treated with 5% for 72 hrs. *P = 0.05 healthy *versus* HCM, *n* ≥ 66 cells. (**C**) Treated with 20% serum for 72 hrs. *P = 0.05 healthy *versus* HCM, *n* ≥ 64 cells. (**D**) Cell surface area of hiPSC-CM ±100 μM PE for 72 hrs. *P = 0.05 no PE added *versus* PE-treated, *n* ≥ 50 cells. (**E**) Cell surface area of hiPSC-CM treated with 5% or 20% serum for 7 days ±100 μM PE for 72 hrs, *n* ≥ 39 cells. (**F**–**H**) Stacked bar graph of the percentage of cells of different sarcomeric structural class (visible well-organized striations (+; category 1), some (disorganized) striations (+/−; category 2), and poor/non-existent striations (−; category 3)); F: no serum; G: treated with 5% serum; H: treated with 20% serum; *n* ≥ 47.

Since hiPSC-CM from patients with gene mutations affecting the heart are now being increasingly used to model cardiac disease, it is important to determine whether serum, still widely used in the culture of cardiomyocytes, would affect the measurement of the disease phenotype in culture. To address this, we used a series of control and disease hiPSC lines derived in our laboratory (characteristics shown in Fig. S3A and B), differentiated them to cardiomyocytes and assessed the impact of exposure to serum or adrenergic agonists on cell surface area and automaticity. hiPSC-CM derived from a healthy individual and three patients with a mutation in the MYBPC3 gene linked to HCM (Fig. S3C) were kept in serum-free medium and were treated with 5% or 20% serum or left untreated for 3 or 7 days. After 3 days, HCM-iPSC-CM, in serum-free medium, was significantly larger than the ctrl-hiPSC-CM, with a ≥1.5-fold increase in cell surface area (Fig. 4A). The healthy control hiPSC-CM displayed a threefold increase in surface area on exposure to either 5% or 20% serum (Fig. 4B and C). The hiPSC-CM from the HCM disease patients were significantly larger than the control in serum-free conditions, suggesting that they had become hypertrophic without additional stimuli, but these cells failed to show a further increase in surface area in response to serum (Fig. 4A–C). Similarly to the hESC-CM under serum-free conditions, the surface area of the control hiPSC-CM increased following 72 hrs exposure to 100 μM PE (Fig. 4D), whereas the hiPSC-CM from the three disease patient lines showed no response (Fig. 4D and Fig. S4). 100 nM ISO had no effect (Fig. S4).

Long-term (>7 days) exposure of hiPSC-CM to serum produced similar results as short-term exposure (72 hrs): the healthy control hiPSC-CM showed an increase in cell surface area while the HCM-hiPSC-CM showed no change upon serum exposure (Fig. 4E). Reflecting the hESC-CM results in Figure 2B, hiPSC-CM also failed to show an additional hypertrophic response to PE when maintained in serum (Fig. 4E).

Upon examination of the sarcomeric structure of the hiPSC-CM, the percentage of cells in category 3 was increased after 72 hrs treatment with serum only in the healthy control hiPSC-CM while the percentage of category 3 cells was unchanged in the HCM-hiPSC-CM (Fig. 4F–H).

## Discussion

There are numerous reports documenting the adverse effects of serum on the efficiency of cardiac differentiation of PSCs [34,35]. Nevertheless serum is often used as a standard medium additive to improve cardiomyocyte viability both during and after differentiation by providing growth factors, nutrients and hormones [19]. However, the effect of serum on cardiomyocytes during their long-term maintenance in culture has not been investigated in much depth even though several studies have described hypertrophic effects of serum on primary rodent cardiomyocytes in culture [36,37]. For this reason, serum concentrations are usually reduced or absent in experiments carried out on these cells. In this study, we first confirmed the published effects of serum and PE on primary neonatal Rat-CM and then investigated the effects of serum on cardiomyocytes derived from hESC and hiPSC, both from a healthy individual and patients with a mutation in the MYBPC3 gene causing HCM. We found that the hypertrophic and physiological effects on the Rat-CM were as reported but the effects on the hPSC-derived cardiomyocytes varied depending on how long they were exposed to serum (72 hrs or >7 days) and which concentration had been present. Hypertrophic and physiological effects of serum were only seen in healthy hPSC and not in cardiomyocytes from the hiPSC lines derived from patients carrying the HCM associated mutation.

hESC-CM and hiPSC-CM derived from the healthy individual showed similar responses to serum, with an increased cell surface area and altered beat rate. They were also similar in their responses to PE and ISO. However the hiPSC-CM derived from patients with a mutation causing HCM showed no response to the addition of serum; they were larger than controls at the outset in serum-free conditions and this did not increase further. These cells were also unresponsive to PE and ISO. The underlying mechanism for this is not clear but is being investigated in an independent study.

In the hESC-CM and healthy hiPSC-CM, short-term serum (72 hrs) exposure resulted in similar changes in cell surface area and beating frequency as PE, a known adrenergic agonist and inducer of hypertrophy in cardiomyocytes *in vitro*. This effect was not reversed by the removal of serum, even after 10 days. In addition, ISO had no effect on the beating frequency of hPSC-CM exposed to serum while in the absence of serum cells doubled their beating frequency as expected (Fig. 2D and E). Our results suggest serum may control cardiac hypertrophy through the adrenergic pathway while additional unknown components in serum, and not present in the PE or ISO conditions, could cause the adverse effects on the sarcomeric structure. In concordance, previous studies demonstrated that isolating and maintaining Rat-CM in serum-free medium resulted in a decrease in stress-like fibres which are more commonly seen in cardiac non-muscle cells [21]. Moreover, studies in isolated human and chick cardiomyocytes have demonstrated the presence of stress-fibres both in unhealthy [38] and immature [39] cardiomyocytes. On the other hand, it is possible that the breakdown of sarcomere structure may only be remodelling of cell structure because of chronotropic or inotropic changes caused by unknown components in the serum. However, the complexity of the adrenergic pathway and the undefined factors in serum require additional investigation. Previous studies on Rat-CM have revealed various parts of the hypertrophy pathway with differing responses to serum [36,37,40]. In one report, the endothelin 1 hypertrophic pathway was suppressed by retinoic acid but serum-induced hypertrophy was not suppressed [37]. In a later study PE and serum-induced hypertrophy were both found to be impaired by the overexpression of CHAMP [40]. Taken together with our results and the lack of an effect of ISO on the beating frequency of hESC-CM after long-term exposure to serum, it might be inferred that serum affects the same pathway as β-adrenergic stimuli rather than α-adrenergic stimuli.

When hESC-CM or healthy hiPSC-CM were treated with serum for longer periods of time (> 7 days) they also showed an increase in cell surface area but lost their augmented response to PE. While other drugs were not tested, these results implied that serum in cell cultures could mask, or otherwise alter, drug-induced effects. Ren *et al.,* for example, showed glucocorticoids induced hypertrophy in rat embryonic cardiomyocytes (H9C2 cells) cultured in serum but in the absence of serum, glucocorticoids protected the cardiomyocytes from apoptosis. Furthermore, this increased cell surface area reverted when the serum was removed for at least 5 days [23]. However the exact cause of this phenomenon is unclear. It may be that the sudden withdrawal of growth factors from the serum leads to problems in basic cell metabolism and the synthesis and trafficking of sarcomeric proteins has become comprised. Thus, the cardiomyocytes with longer exposure to serum may be on their way to losing viability. Others have previously reported similar results in Rat-CM in which serum-deprivation lead to increased apoptosis [41].

In addition to PSC-CM from healthy individuals, we investigated the effects of serum on cells derived from three patients with HCM caused by a mutation in MYBPC3 gene. Clinically these patients had an increase interventricular septum thickness but other heart functions, such as fractional shortening, LV systolic-end and diastolic-end diameter, remained within the normal range [42]. Their derivative hiPSC-CM was resistant to PE and ISO induced hypertrophy and chronotropic effects and they also lacked the response to serum seen in the controls and primary cardiomyocytes. Thus their enlarged cell surface areas and altered beat rates relative to controls in the absence of serum were no longer evident in the presence of serum. This represents a cautionary note on the culture conditions used to compare diseased and control cardiomyocytes derived from pluripotent stem cells. In addition, some experiments described in the literature use primary rodent cells ±24 hrs after transfer to serum-free medium but this may not always be the case. Improved consistency in the results may be achieved by carrying out the experiments under serum-free or low serum conditions.

In conclusion, we have demonstrated that the addition of serum to PSC-CM derived under defined and conventional serum-free conditions can significantly alter the phenotype of cardiomyocytes: their surface area, sarcomeric structure and beating frequency are all parameters that show relevant alterations. These are among the parameters widely used to report phenotypes in cardiomyocytes derived from patient hiPSC. Therefore it is highly desirable to control experimental conditions and to culture hPSC-derived cardiomyocytes in fully defined culture media.

## Supporting information

**Figure S1** Cardiac differentiation efficiency of the Nkx 2-5^eGFP/w^ hESC line and LUMC0004ictrl determined by flow cytometry of cardiac troponin T (cTnT) expression (*n* = 11 for Nkx 2-5^eGFP/w^, two independent experiments for LUMC0004ictrl. **Figure S2** Stacked bar graph of the percentage of RAT-CM with sarcomeric structural class (visible well-organized striations (+; category 1), some (disorganized) striations (+/−;category 2), and poor/non-existent striations (−;category 3)); *n* ≥ 41 cells. **Figure S3** Characterization of hiPSC. **Figure S4** Response of hiPSC-CM to isoproterenol (ISO).
