# Supplementary material for: Serum supplemented culture medium masks hypertrophic phenotypes in human pluripotent stem cell derived cardiomyocytes
Source: J Cell Mol Med. 2014 Jul 1;18(8):1509–18. doi: 10.1111/jcmm.12356 (PMC4190898; doi:10.1111/jcmm.12356)
Supplement: Supplementary file 1 [file jcmm0018-1509-SD1.doc]

**Supporting Information**

**Serum supplemented culture medium diminishes hypertrophic phenotypes in human pluripotent stem cell derived cardiomyocytes**

**Cheryl Dambrot, Stefan R. Braam, Leon G. J. Tertoolen, Matthew Birket, Douwe E. Atsma, Christine L. Mummery**

**Material and Methods**

*Transgene removal and confirmation*

Based on morphology, hESC-like colonies were manually picked 30 days after transduction. Cells were adapted to enzymatic culture on mouse embryonic feeder cells [1] and then transfected with pLV.hCMV-IE.FLPe.IRES.PurR.hHBVPRE [2] to remove the reprogramming cassette.

Removal was confirmed by PCR of the FRT region (triple primer set): 5’-CGAGTCGGATCTCCCTTTGGGC-3’, 5’-TGGAAGGGCTACGTAGCTAGC-3’, 5’-GGTTCCCTAGTTAGCCAGAGAGC-3’). Transgene-free clones were readapted to mechanical passage on Matrigel with mTESR. Frozen stocks were made at passage 5 and cells were used for experimentation between passage 10 and 30.

### *Immunostaining*

### Immunofluorescent staining was performed as previously [3]. In brief, PFA-fixed cells were permeabilized with Triton X-100, blocked with 4% normal goat serum for 1 h before overnight incubation with the primary antibody (NANOG (1:500, Peprotech), SSEA-4 (1:30, Biolegend), OCT3/4 (1:100, Santa Cruz), TRA-1-81 (1:25, Biolegend), βIII-TUBULIN (1:2000, Covance), AFP (1:25, Quarttet), CD31 (1:100, DAKO) at 4 °C. The next day, secondary antibodies labeled with Cy3 (1:200, Jackson Immuno Research), Alexa 568 (1:200, Invitrogen) or Alexa 488 (1:500, Invitrogen) were added for 1 h at room temperature. Nuclei were stained with DAPI before mounting slides with Mowiol (Calbiochem).

*Karyotype analysis*

Karyotype analysis was performed using COBRA-FISH as previously described [4]. 20 metaphase spreads for each sample were analyzed.

*Mutation Confirmation*

DNA from hiPSC was collected using a phenol chloroform-based method. MYBPC3 exon 25 was amplified by PCR (forward primer: 5’-cctgtggcggttagttgg-3’, reverse primer: 5’ caccggtagctcttcttcttcttg-3’) and used for Sanger sequencing with the forward primer.

*Spontaneous Differentiation of hiPSC into derivatives of the three germ layers*

Pieces of hiPSC colonies were passaged onto Matrigel coated chamber slides (BD Falcon) in mTeSR for two days, then cultured in DMEM/F12 with 20% FCS for 3 weeks, changing medium every other day. Cells were then fixed for 30 minutes in 2% paraformaldehyde (PFA).

References

[1] **Dambrot C, Van Den Berg C, Oostwaard DW et al.** Chapter 27 - Cardiomyocyte Differentiation of Human Pluripotent Stem Cells. In. Human Stem Cell Manual (Second Edition). Boston: Academic Press; 2012; p. 413-31.

[2] **Goncalves MA, Janssen JM, Holkers M, de Vries AA**. Rapid and sensitive lentivirus vector-based conditional gene expression assay to monitor and quantify cell fusion activity. *PLoS One* 2010;5:e10954.

[3] **Davis RP, Casini S, van den Berg CW et al.** Cardiomyocytes derived from pluripotent stem cells recapitulate electrophysiological characteristics of an overlap syndrome of cardiac sodium channel disease. *Circulation* 2012;125:3079-91.

[4] **Szuhai K, Tanke HJ**. COBRA: combined binary ratio labeling of nucleic-acid probes for multi-color fluorescence in situ hybridization karyotyping. *Nat Protoc* 2006;1:264-75.

**
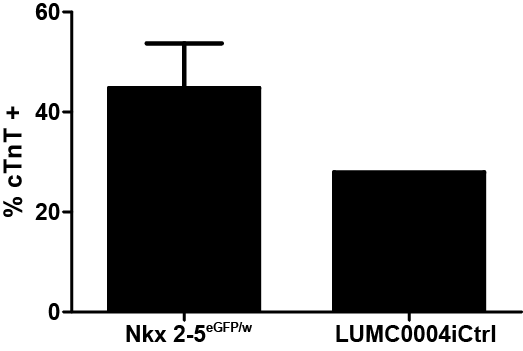
**

Fig S1) Cardiac differentiation efficiency of the Nkx 2-5^eGFP/w^ hESC line and LUMC0004ictrl determined by flow cytometry of cardiac troponin T (cTnT) expression (n=11 for Nkx 2-5^eGFP/w^, two independent experiments for LUMC0004ictrl.

**
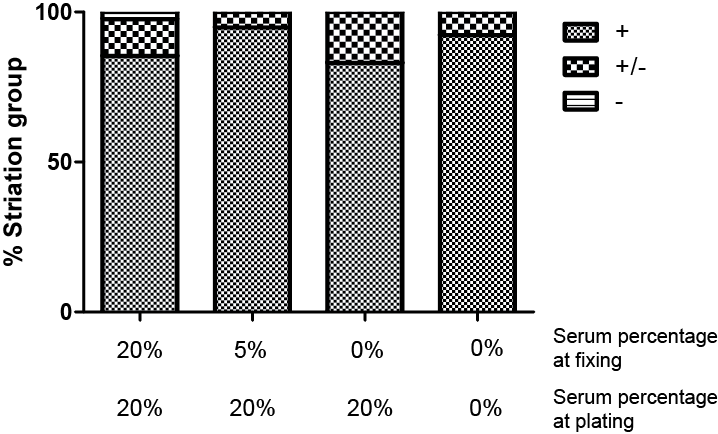
**

Fig S2) Stacked bar graph of the percentage of RAT-CM with sarcomeric structural class (visible well-organized striations (+; category 1), some (disorganized) striations (+/-;category 2), and poor/non-existent striations (-;category 3)); n≥41 cells.

**
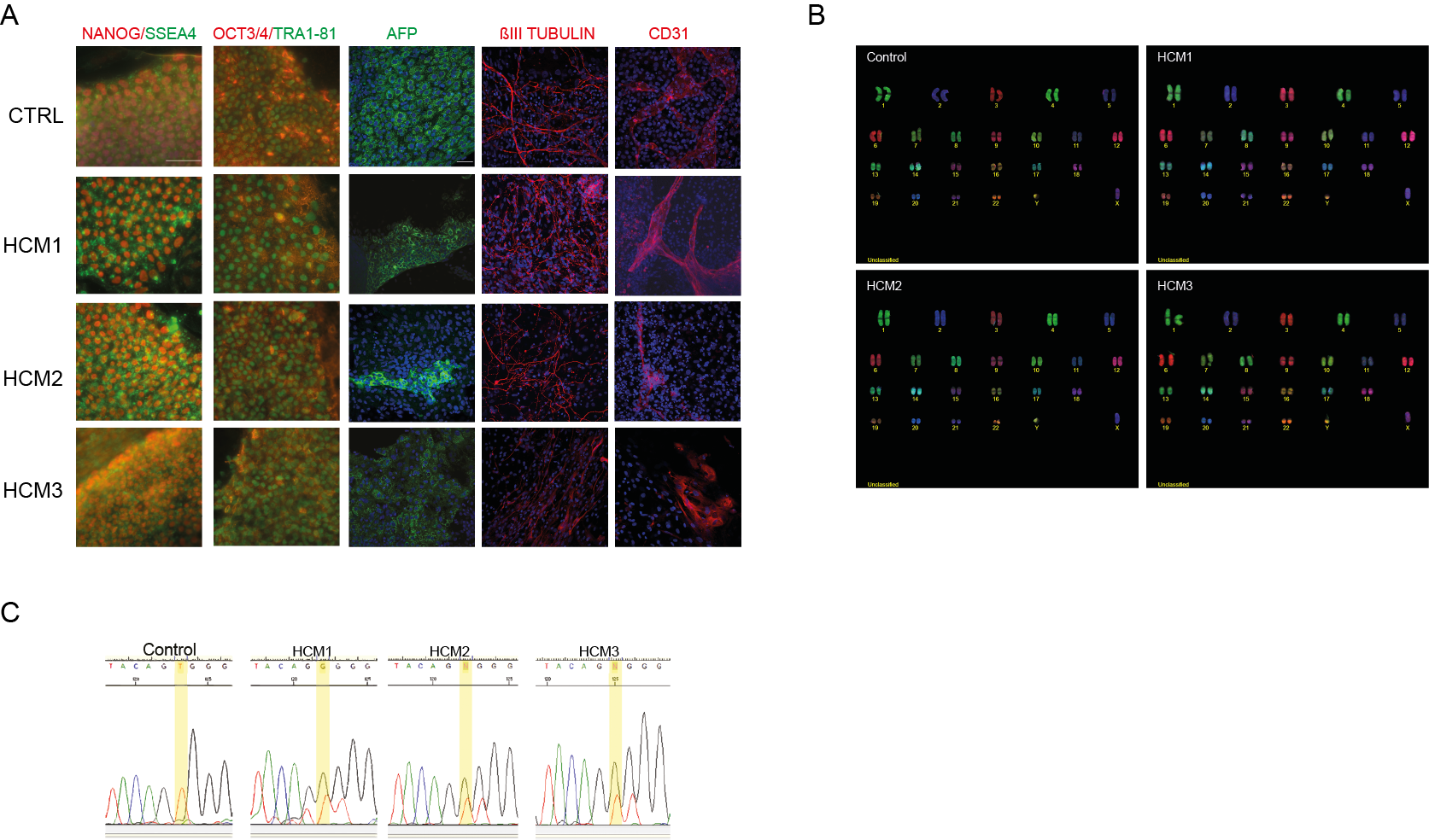
**

Fig S3: Characterization of hiPSC. **A)** Immunofluorescence images of undifferentiated hiPSC expressing typical pluripotency markers (red: NANOG, TRA1-81; green: OCT3/4, SSEA-4) and spontaneously differentiated hiPSC stained for derivatives of the three germ layers (alpha feto-protein (AFP, green); βIII-Tubulin (red); CD31 (red); nuclei (DAPI, blue). Scale bar: 50µm). **B)** Karyogram generated by combined binary ratio labeling fluorescence *in situ* hybridization (COBRA-FISH) of HCM and ctrl hiPSC showing a normal 46XY karyotype. **C)** Sanger sequencing results of exon 25 of MYBPC3 of control and HCM-iPSC.


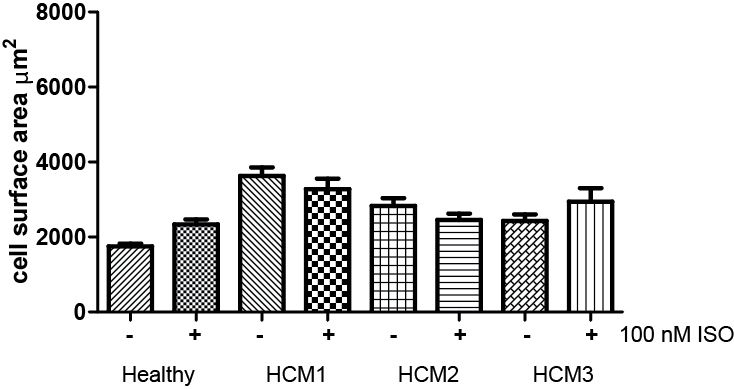


Fig S4: Response of hiPSC-CM to isoproterenol (ISO). Cell surface area of hiPSC-CM ± 100 nM ISO for 72 h (n≥25 cells).
